# Microbial diversity in active and abandoned desert kangaroo rat burrows and from proximal surface sand

**DOI:** 10.1128/spectrum.01388-24

**Published:** 2025-04-08

**Authors:** Duygu Aydin, Janice M. Parks, Sera Tirkes, Clint E. Collins, Idil Deniz Akin, Maren L. Friesen, Douglas R. Call, Haluk Beyenal

**Affiliations:** 1 School of Chemical Engineering and Bioengineering, Voiland College of Engineering and Architecture Washington State University 184730 , Pullman, Washington, USA; 2 Department of Plant Pathology, Washington State University 6760, Pullman, Washington, USA; 3 Department of Civil and Environmental Engineering, University of California Los Angeles 8783, Los Angeles, California, USA; 4 Department of Biological Sciences, Sacramento State University 173226, Sacramento, California, USA; 5 Paul G. Allen School for Global Health, Washington State University 6760, Pullman, Washington, USA; Nanjing Agricultural University, Nanjing, China

**Keywords:** kangaroo rat burrow, community analysis, microbial diversity, 16S rRNA, biofilm

## Abstract

Desert kangaroo rats (*Dipodomys deserti*) construct burrows that can create micro-niches favorable to increased microbial activity. The aim of this study was to characterize the bacterial communities found in kangaroo rat burrows, in proximal desert surface sand, and in samples from kangaroo rats. We collected samples from burrow ceilings of actively inhabited burrows, from burrows that were no longer in use, and from the proximal surface sand in the Sonoran Desert, Yuma, AZ. Following DNA extraction from samples, 16S rRNA gene sequencing was performed, and functional predictions were made and assessed for each characterized bacterial community. Active burrow samples exhibited greater alpha diversity but similar beta diversity when compared to surface sand (*P* < 0.05), with no significant differences observed between abandoned and active burrows. Bacterial genera and genes related to nitrogen fixation, nitrification, and urea hydrolysis were found in significantly higher abundance in active burrows compared to the surface sand (*P* < 0.05). The core microbiome of active burrow samples was different from surface sand, including higher abundances of *Acidimicrobiales* and *Acidobacteria* subdivision Gp7. Active burrow samples included 30 unique genera. Kangaroo rat anal swabs shared 12, cheek pouches shared 6 unique genera with burrows. These findings suggest that kangaroo rats can shape the microbial composition of their burrow environment through the introduction of food material and waste, facilitating increased species richness and bacterial diversity.

**IMPORTANCE:**

Animals can alter soil parameters, including microbial composition through burrowing activities, excretion, and dietary composition. Desert kangaroo rats (*Dipodomys deserti*) construct burrows within loose desert sand that have microclimatic conditions different from the surrounding desert climate. In this study, we explored the effect of disturbance from kangaroo rat activities on the bacterial composition of sand. We compared the bacterial community compositions of kangaroo rat (*D. deserti*) samples, their burrows, and the proximal surface sand. The results showed that burrow sand shows higher richness and diversity of bacterial community with higher abundances of bacterial genera and genes associated with nitrogen fixation, nitrification, and urea hydrolysis compared to the surface sand. These findings suggest that kangaroo rats affect the microbial composition of their burrow environment through the introduction of food material and waste.

## INTRODUCTION

Desert kangaroo rats construct burrows within loose desert sand that serve as shelters and food storage sites. Kangaroo rats feed predominantly on desert bush seeds, which they collect and transport in their cheek pouches for storage within the burrows (1). Despite the harsh environmental challenges such as rainfall events, storms, and temperature fluctuations, the burrows remain intact over extended periods (2). Burrowing activities of animals can alter soil parameters, including organic matter content, soil moisture, and microbial composition (3, 4). Previous studies have shown that kangaroo rat burrows contain higher levels of nitrates and salts than non-burrow sites at the same depths (5–7). In addition, the burrows have microclimatic conditions that differ from the surrounding desert climate. For example, the temperatures inside the burrows remain persistently stable at moderate levels throughout the day, and the relative humidity reaches 100% inside closed burrows (8, 9). Together with available organic matter and moderate environmental conditions, kangaroo rat burrows provide a favorable environment for increased microbial activity. For example, various fungal species have been identified in kangaroo rat burrows and are known to be derived from the kangaroo rat feet and cheek pouch (1). However, to date, less is known about bacterial community composition in these burrows.

We compared the bacterial community compositions of kangaroo rat (*Dipodomys deserti*) samples, their burrows, and the proximal surface sand. We expected that active burrows would be characterized by higher microbial abundance and a microbial community structure distinct from that of surface sand. Therefore, we designed our work to answer the following questions: (i) what is the overall bacterial composition of kangaroo rats (mouth, cheek, foot, anal swabs), their burrows, and the proximal surface sand; (ii) how do the bacterial communities differ between microbiomes of kangaroo rats, their burrows, and the proximal surface sand; (iii) do kangaroo rat burrows support more microorganisms compared to proximal surface sand; and (iv) are kangaroo rats altering the soil microbiome through the transfer of microorganisms from their own microbiome to their burrows? Samples were collected from the ceilings of kangaroo rat burrows, proximal surface sand, and kangaroo rats in the Sonoran Desert, Yuma, AZ. Following DNA extraction, 16S rRNA gene sequencing was conducted to evaluate the bacterial diversity and community structures of kangaroo rats, their burrows, and the proximal surface sand, with functional predictions made and assessed for each characterized bacterial community.

## RESULTS

### Overall bacterial composition and core microbiome

**Fig 1:**
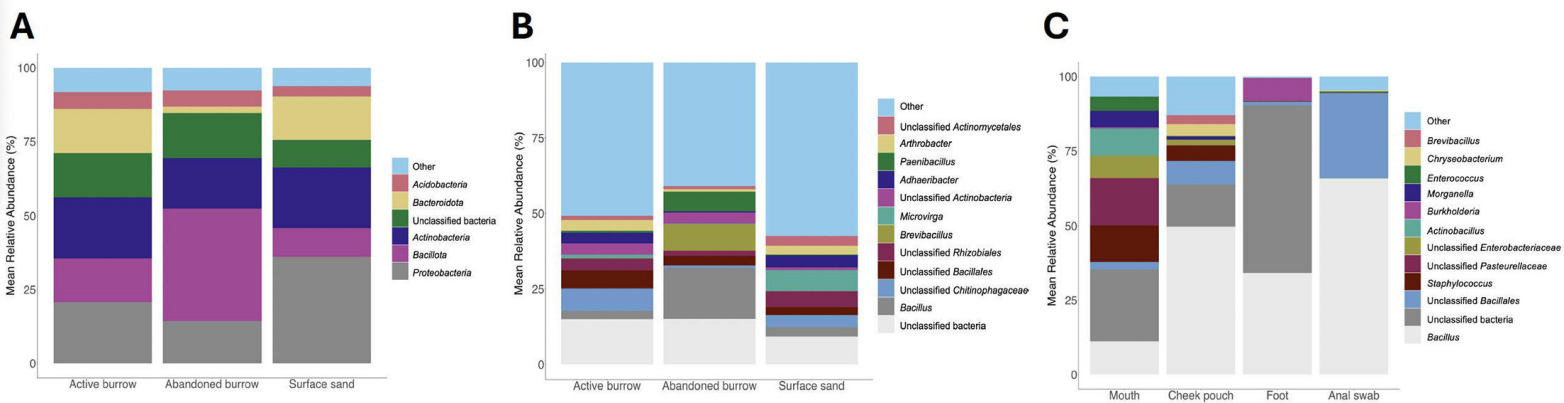
Stacked bar chart of mean relative abundance data (%) (A) active burrow, abandoned burrow, and surface sand community type on phylum level (B) genus level (C) kangaroo rat (mouth, cheek pouch, foot, anal swab) mean relative abundance data (%) on genus level.

The taxonomic composition of samples collected from three distinct active burrows (*n* = 3), three distinct abandoned burrows (*n* = 3), and surface sand (*n* = 3) from three distinct locations was characterized using 16S rRNA sequencing (Fig. 1A and B). Initially, a total of 204,348 sequences were obtained across all samples, all of which were unique. After quality control to reduce Polymerase chain reaction (PCR) and sequencing errors, 18,048 unique sequences and a total of 166,557 sequences were retained across all samples. A total of 18,050 amplicon sequence variants (ASVs) were detected in all sand samples, with most bacterial phyla and genera occurring in low abundance. Among the 22 phyla detected, 16 had mean relative abundances <3%. *Actinomycetota* (formerly *Actinobacteria), Pseudomonadota* (formerly Proteobacteria)*, Bacteroidota* (formerly *Bacteroidetes*), and *Bacillota* (formerly *Firmicutes*) dominated the sand samples. Out of 351 observed genera, only 12 were found to exceed the 3% mean relative abundance. Active burrow bacterial communities included a higher proportion of *Adhaeribacter* and to a lesser extent *Arthrobacter* and *Bacillus. Azotobacter*, *Flavobacterium*, *Clostridium*, and *Nitrospira* were some of the genera found in significantly higher abundance compared to surface sand (Kruskal-Wallis sum-rank test, *P* < 0.05). Furthermore, overall taxonomic composition of animal samples (mouth, cheek pouch, foot, and anal swab) (Fig. 1C) showed high proportions of *Staphylococcus* and *Bacillus*.

The core microbiome (ASVs comprising ≥0.1% of sequences present in 100% of tested samples) of active burrow was comprised of *Bacillus*, *Sporosarcina*, and *Adhaeribacter*, while the core microbiome of surface sand included *Bacillus*, *Microvirga*, unclassified *Rhizobiales*, and *Corallococcus*. No taxa were represented by ≥0.1% of sequences for abandoned burrow (Table 1).

**TABLE 1:** Sample location, core microbiome members (ASVs classified into genera), mean relative abundances (%), and confidence interval

Sample location	Core microbiome member	Mean relative abundance percentage	95% Confidence interval
Active burrow	*Bacillus*	2.82%	0.015–0.041
	*Sporosarcina*	1.8%	0.009–.027
	*Adhaeribacter*	3.55%	0.029–0.042
Surface sand	*Bacillus*	3.15%	0.03–0.034
	*Microvirga*	6.98%	0.060–0.079
	*Unclassified Rhizobiales*	5.27%	0.045–0.06
	*Corallococcus*	1.84%	0.017–0.02

### Alpha and beta diversity

**Fig 2:**
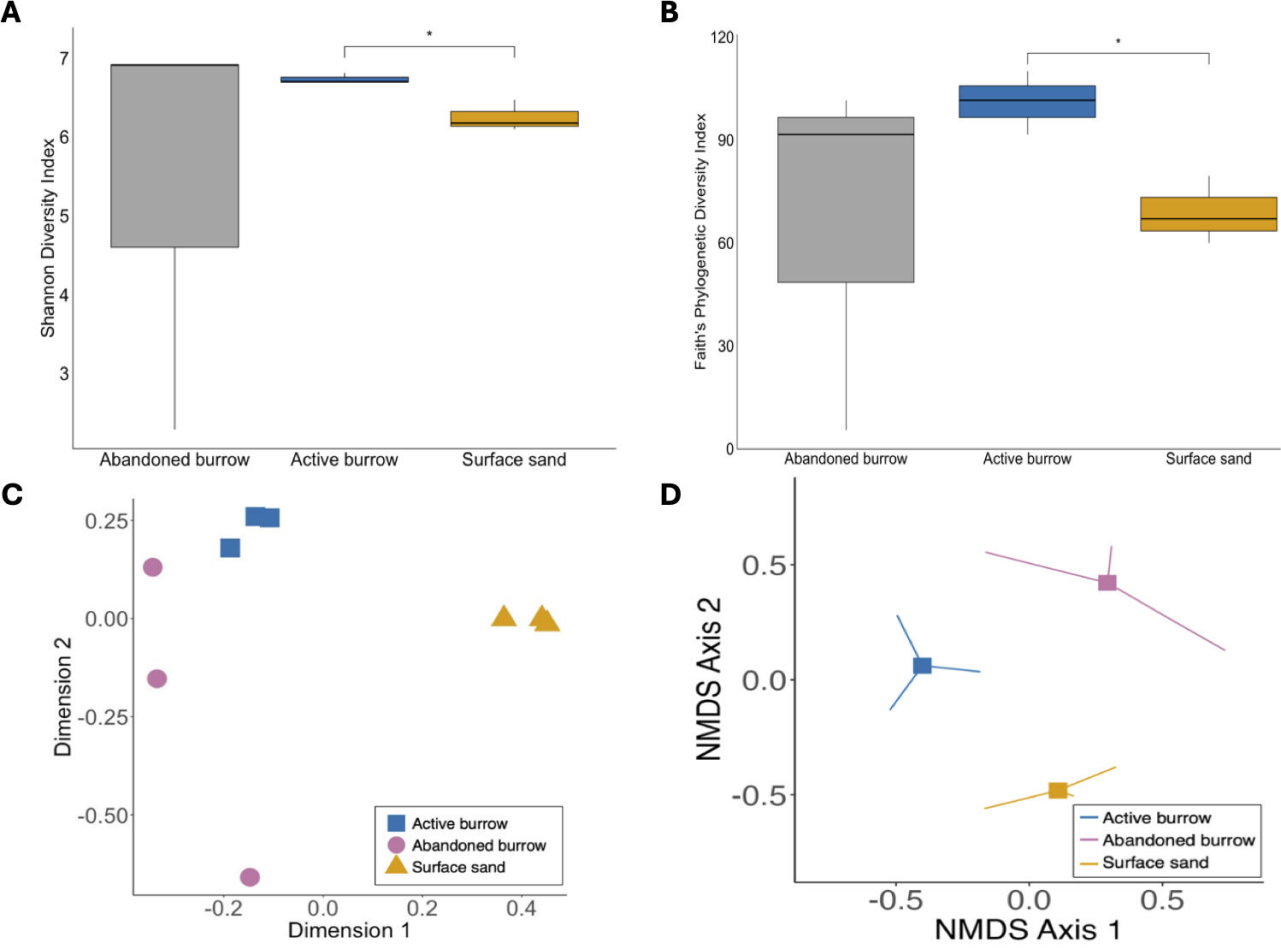
Alpha diversity, measured in terms of (A) Shannon diversity index (B) Faith’s phylogenetic diversity index of active burrow, abandoned burrow, and surface sand. The upper and lower boundaries of each box represent the 75th and 25th quartile values. The lines inside the boxes show the median values. The ends of the lines indicate the lowest and highest diversity values. Active burrow and surface sand showed significant differences (*P* < 0.05) based on the Kruskal-Wallis sum-rank test. Bray-Curtis distance principal coordinates analysis (PCoA) show clustering of ASVs in active burrow (square), abandoned burrow (circle), and surface sand (triangle) (C). Bray-Curtis distance nonmetric multidimensional (NMDS) ordination plot of microbial communities in active (blue), abandoned burrow (pink), and surface sand (yellow) based on bacterial ASVs. Each line shows the number of samples analyzed, and the squares represent the centroids (D). ASV, amplicon sequence variant.

The measures of alpha diversity both in terms of Shannon diversity index and Faith’s phylogenetic diversity showed significant differences between active burrow and surface sand (Kruskal-Wallis sum-rank test, *P* < 0.05) (Fig. 2B). Active burrow samples had higher diversity and evenness of microbial community compared to the surface sand, as indicated by the Shannon and Faith’s Phylogenetic Diversity indices (6.8 and 101.0 for active burrow and 6.3 and 68.8 for surface sand, respectively). However, no significant differences were found between active burrow and abandoned burrow comparisons or for surface sand and abandoned burrow comparisons. The abandoned burrow showed more deviation. The measures of beta diversity, Bray-Curtis, and UniFrac distances (both weighted and unweighted) exhibited no significant differences between active burrow and surface sand (*F* = 3.37, *P* = 0.09 for Bray-Curtis), (*F* = 7.32, *P* = 0.09 for weighted UniFrac distances), and (*F* = 1.88, *P* = 0.09 for unweighted UniFrac distances), and between abandoned burrow and active burrow (*F* = 1.51, *P* = 0.088 for Bray-Curtis), (*F* = 4.42, *P* = 0.107 for weighted UniFrac distances), and (*F* = 1.76, *P* = 0.107 for unweighted UniFrac distances). To further compare the communities from different sample locations, nonmetric multidimensional scaling (NMDS) and principal coordinates analysis (PCoA) ordination plots were produced from Bray-Curtis similarity index matrices of all samples (Fig. 2C and D). Although the beta diversity measures did not statistically differ, NMDS and PCoA plots clearly differentiated the microbial communities belonging to active burrow and surface sand consistent with our hypothesis that active burrows support a distinct microbial community structure compared to surface sand. In contrast, high dissimilarities among abandoned burrows were seen.

### Differentially abundant and unique bacterial communities

**Fig 3:**
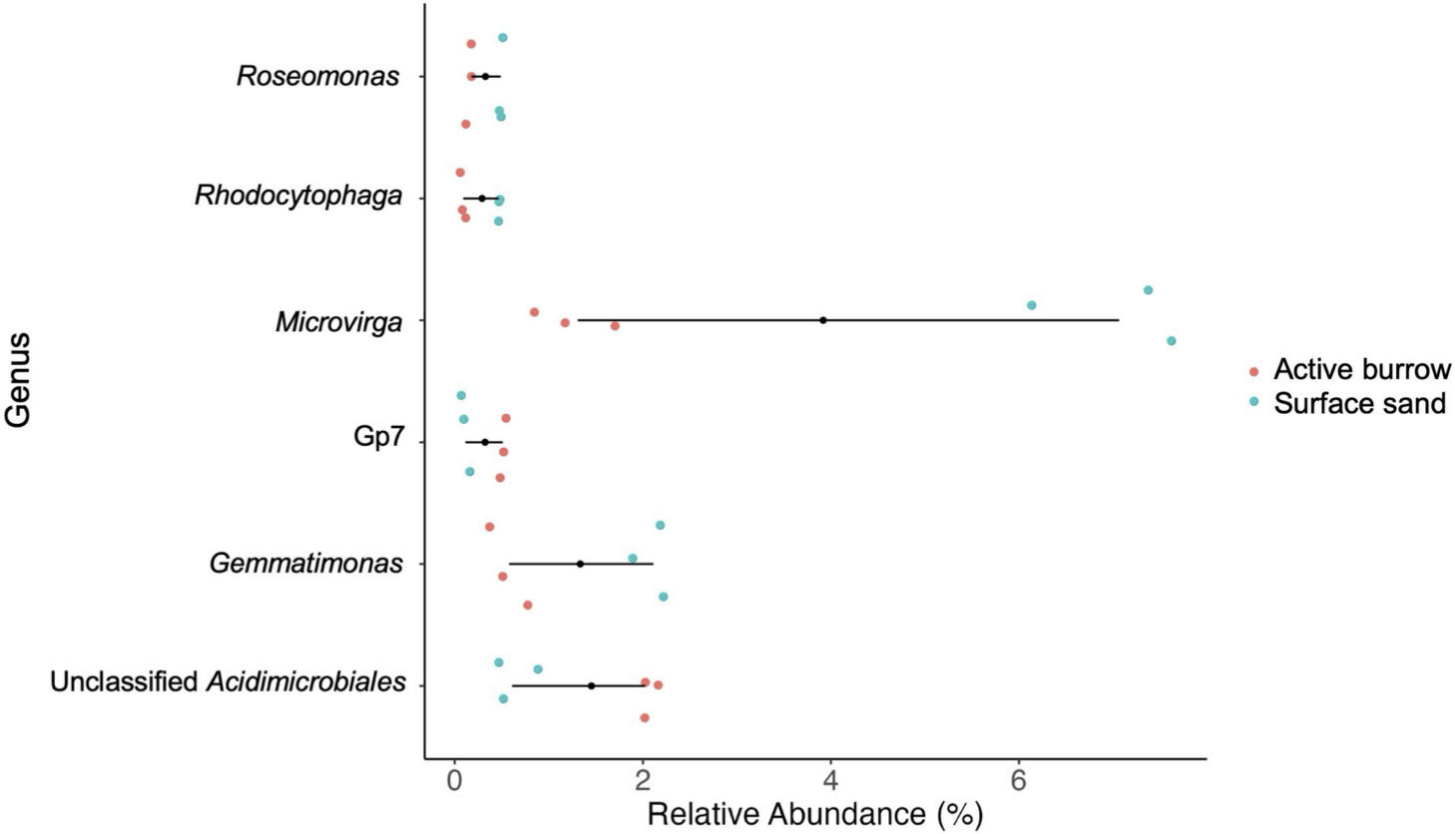
Relative abundance of differentially abundant ASVs classified into genera for active burrow and surface sand. The mean relative abundance is represented for active burrow and surface sand. Their differences are represented by a solid black line. The center black point represents the median point. ASV, amplicon sequence variant.

More abundant bacterial genera from active burrow samples included *Acidobacteria* subdivision Gp7 and unclassified genus of *Acidimicrobiales* (Fig. 3). *Roseomonas*, *Rhodocytophaga*, *Microvirga*, and *Gemmatimonas* were significantly more abundant in surface sand.

**Fig 4:**
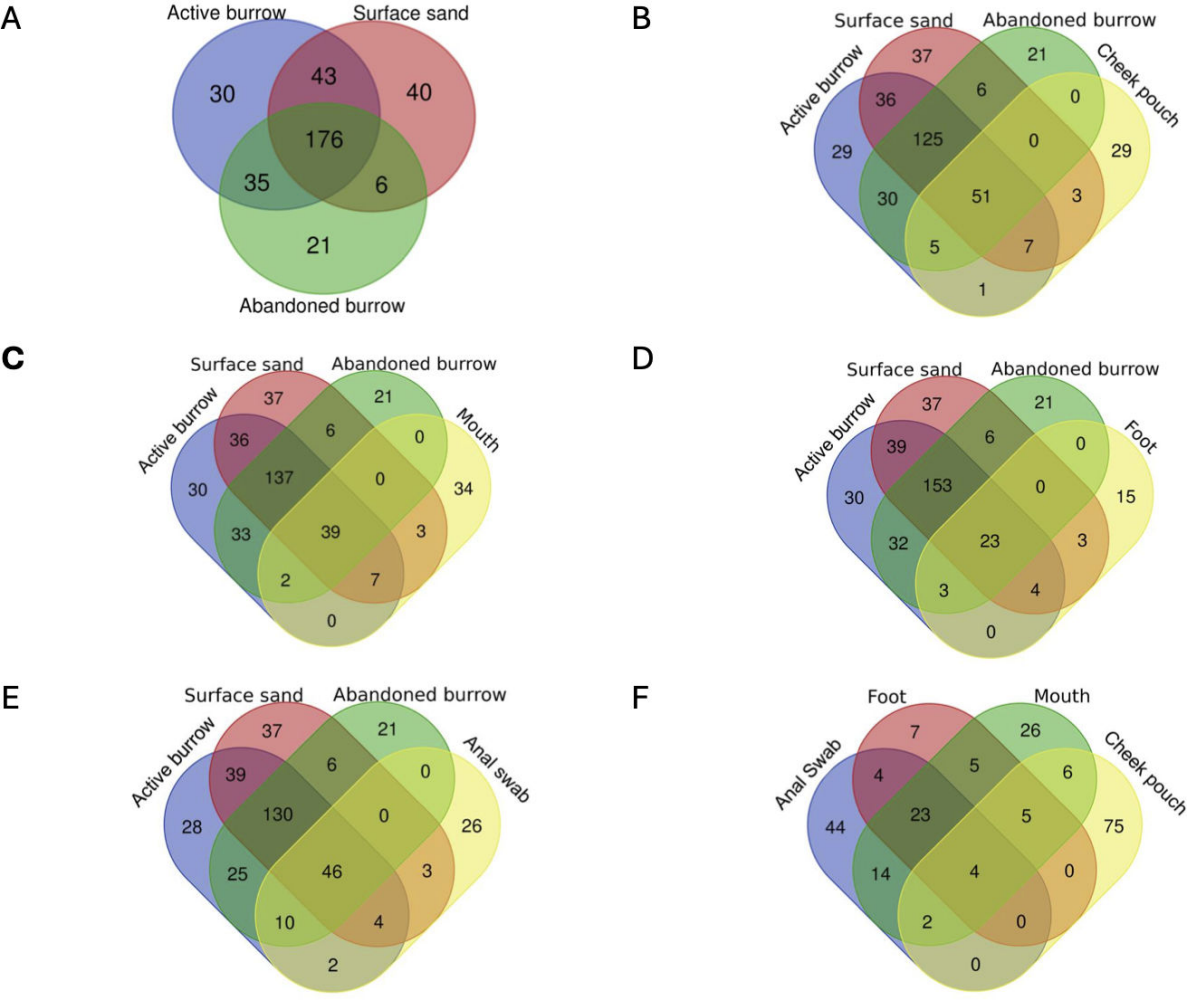
Venn diagrams illustrating the number of shared and unique ASVs classified into genera among (A) active burrow, abandoned burrow, surface sand; (B) active burrow, abandoned burrow, surface sand, and kangaroo rat cheek pouch; (C) active burrow, abandoned burrow, surface sand, and kangaroo rat mouth; (D) active burrow, abandoned burrow, surface sand, and kangaroo rat foot; (E) active burrow, abandoned burrow, surface sand, and kangaroo rat anal swab samples; and (F) kangaroo rat cheek pouch, mouth, foot, and anal swab samples. Thirty genera were unique to the active burrow community. Anal swab and cheek pouch samples shared the highest number of genera with the active burrow microbial community. ASV, amplicon sequence variant.

We analyzed the shared ASVs classified into genera among animal samples (cheek pouch, mouth, foot, and anal swab) and sand samples (active burrow, abandoned burrow, and surface sand) (Fig. 4). Using a Venn diagram, we visualized the overlapping genera among sample groups. Among active burrow, abandoned burrow, and surface sand, 176 overlapping genera were found (Fig. 4A). Thirty genera were found unique to the active burrow community. The genera only present in active burrow were unclassified *Rhizobiales*, *Variovorax*, *Singularimonas*, *Vampirovibrio*, *Actinocorallia*, *Cellulosilyticum*, *Rhodanobacter*, *Turicella*, *Ferruginibacter*, *Rhodoplanes*, *Herpetosiphon*, *Cytophaga*, *Cryptosporangium*, *Aminobacter*, *unclassified Mycobacteriaceae*, *Belnapia*, *Arenimonas*, *Phycicoccus*, *Mucilaginibacter*, *Solimonas*, *Flavihumibacter*, *Pyxidicous*, *Craurococcus*, *Parapedobacter*, unclassified *Deinococcaceae*, unclassified *Alicyclobacillaceae*, *Methylophilus*, and unclassified *Alcaligenaceae.* Cheek pouch samples shared the highest number of genera with the sand samples (10) (Fig. 4B). When considering both active and abandoned burrows and their unique overlap with different animal compartments, we find that anal swabs share 12 unique genera with burrows while cheek pouches share 6 unique genera with burrows (Fig. 4B through E).

### Functional predictions

**Fig 5:**
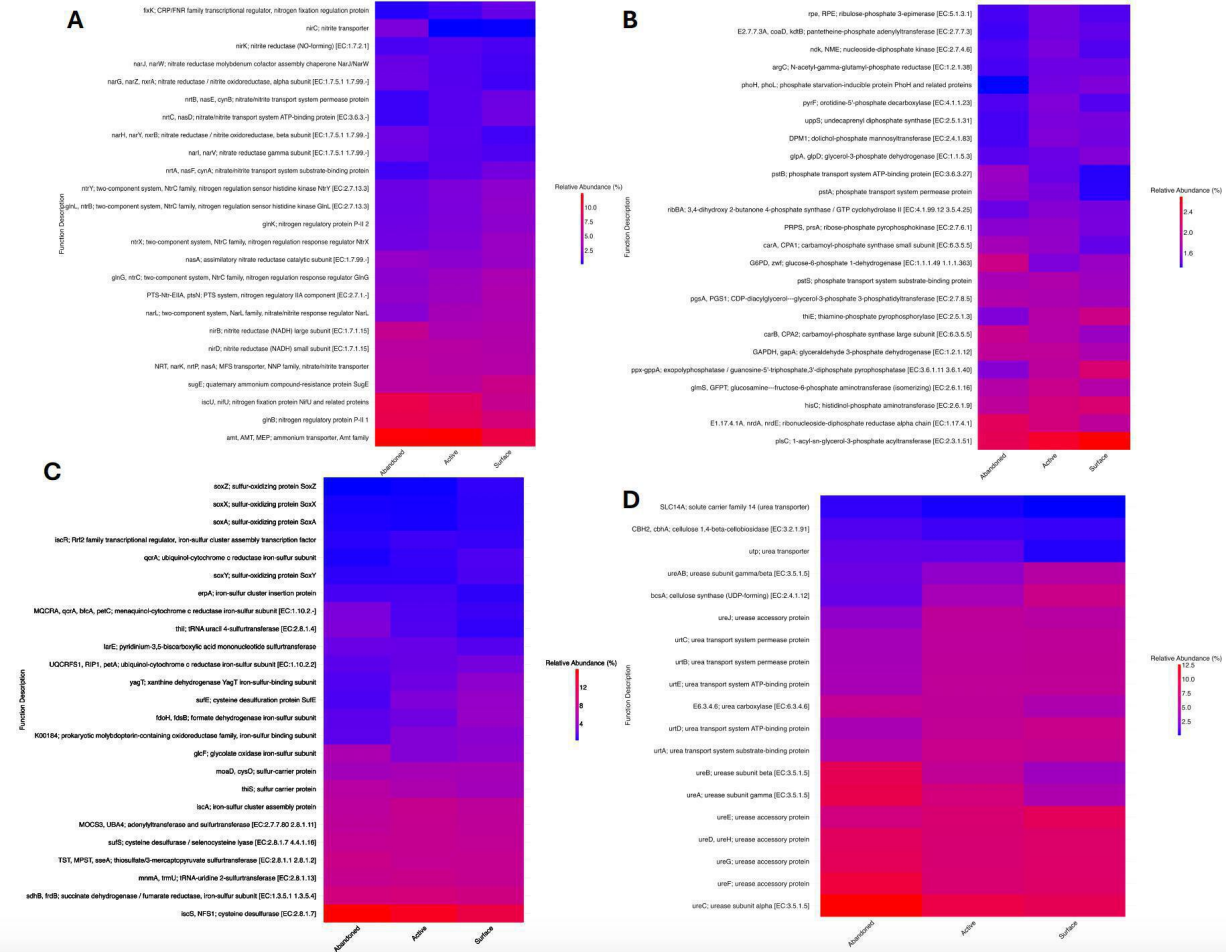
Heatmap showing PICRUSt2 inferred functional relative abundances in active burrows, abandoned burrows, and surface sand predicted using PICRUSt2. Functions are categorized as (A) nitrogen metabolism, (B) phosphate metabolism, (C) sulfur metabolism, and (D) urea and cellulose metabolism.

PICRUSt2 (Phylogenetic Investigation of Communities by Reconstruction of Unobserved States) was conducted to predict functional profiles of microbial communities in active burrows, abandoned burrows, and surface sand from 16S rRNA data. Predicted functional abundance tables of ASVs were created based on the Kyoto Encyclopedia of Genes and Genomes pathways. Key findings in burrow samples included genes associated with nitrogen metabolism, such as those coding for nitrogen fixation (*iscU* and *nifU*), nitrate reduction (*narG*, *narZ*, and *nxrA*), and nitrite reduction (*nirD*). Genes associated with phosphate and sulfur metabolism included those coding for polyphosphate glucokinase (*ppgK*), sulfur carrier protein (*thiS*), cysteine desulfurase (*iscS* and *NFS1*), and thiosulfate sulfurtransferase (*glpE*). Additionally, genes coding for urease accessory proteins (*ureD* and *ureF*) and urease subunits (*ureA*, *ureB*, and *ureC*), as well as urea transporter (*utp*) involved in urea hydrolysis were identified. Genes coding for cellulose synthase (*bcsA*) were also detected in active burrow samples (Fig. 5A through D). Genes involved in nitrogen metabolism pathways, such as ammonium transporter proteins (*amt*), nitrogen fixation proteins (*iscU* and *nifU*), and nitrite reductase (*nirD*) were found in significantly higher abundances in active burrow samples compared to surface sand samples. Similarly, in urea and sulfur metabolism, genes such as urea carboxylase, urease (*ureAB*), and sulfur carrier proteins (*thiS* and *iscS*) were also significantly more abundant in active burrow samples (Wilcoxon test, *P* < 0.05). As PICRUSt2 only predicts gene presence and abundance based on 16S rRNA data, the results presented here require experimental validation in future studies.

## DISCUSSION

**Fig 6:**
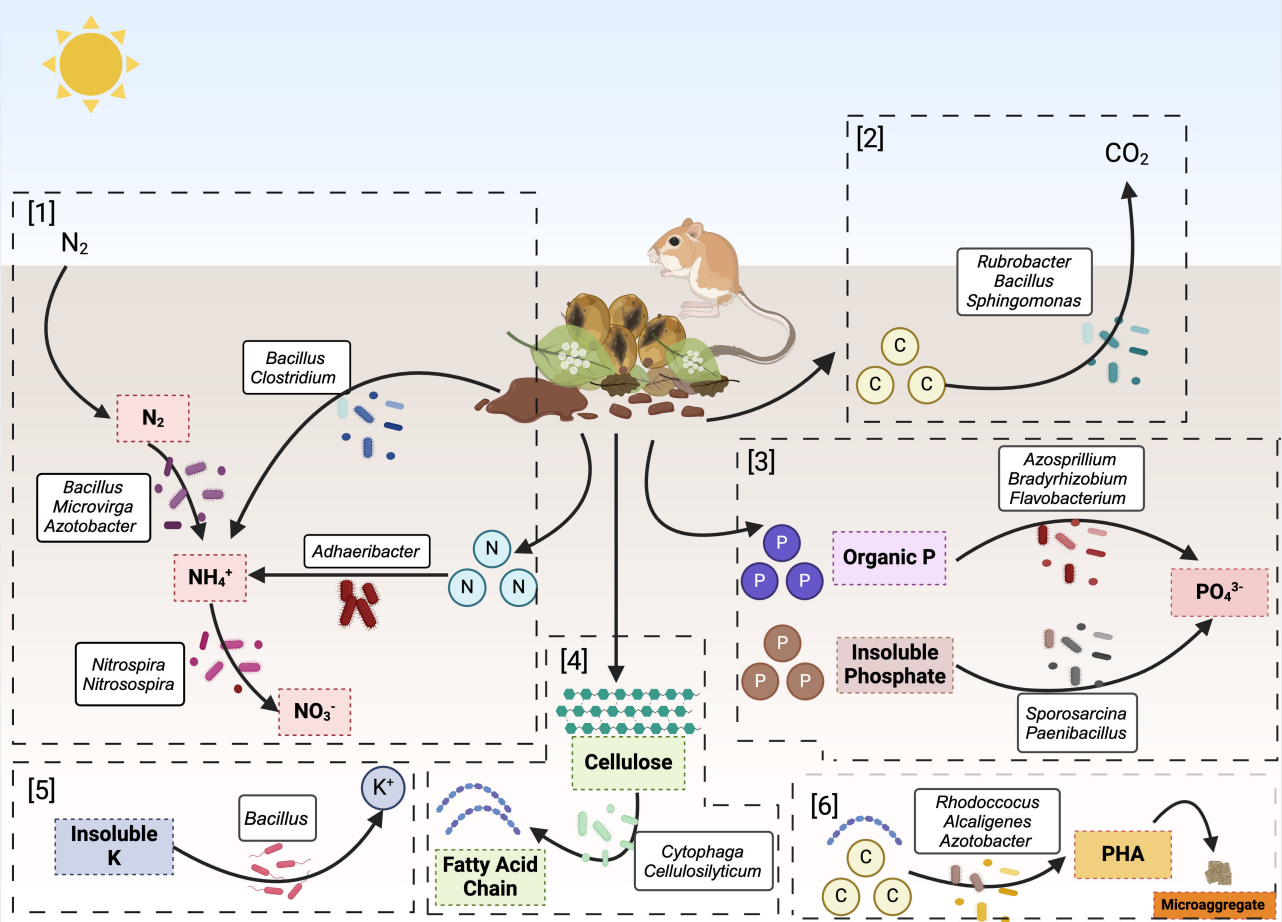
Proposed key burrow nutrient transformations associated with the active burrow microbial community. The figure summarizes the key findings and proposed pathways based on information from the literature and PICRUSt2 analysis. Biological remains, including leaf litter, moldy seeds, and kangaroo rat feces, provide a nutrient source for the abundant bacteria found in active burrows. [1] Nitrogen from the atmosphere is fixed by nitrogen-fixing bacteria. Organic nitrogen compounds are converted into ammonium by ammonifying and urease-active bacteria. Nitrifying bacteria convert ammonium into nitrate. [2] Carbon sources found in biological remains are used as energy sources by carbon-utilizing bacteria. [3] Phosphate-solubilizing bacteria break down organic phosphorus from biological remains. Insoluble phosphate found in active burrow sand is converted into available form of phosphate by phosphorus-solubilizing bacteria. [4] Cellulolytic bacteria hydrolyze cellulose into short fatty acid chains. [5] Insoluble potassium is metabolized by *Bacillus* into available form of potassium. [6] PHA-producing organisms (*Rhodococcus*, *Alcaligenes*, and *Azotobacter*) can contribute to microaggregate formation. Created with BioRender (https://www.biorender.com/).

The alpha diversity measurements, both in terms of Shannon diversity index and Faith’s phylogenetic diversity, revealed significant differences between active burrow and surface sand (*P* < 0.05). The higher alpha diversity indicates that the active burrows had higher diversity and evenness of microbial community compared to the surface sand. This is most likely due to the changes in environmental conditions such as water availability, nutrient content, and temperature in the burrows as a result of burrowing activities of kangaroo rats and the addition of microbes to burrows from animal microbiomes. The environmental conditions influence the diversity and structure of the microbial community found in burrows (11, 12). For instance, animal burrowing activities can influence water availability (13). The burrows of kangaroo rats are reported to have higher ambient humidity (9). Therefore, the water loss to evaporation is limited. This can increase the microbial activity and diversity due to the higher diffusion of minerals, nutrients, and oxygen (14). Additionally, nutrient-rich microsites are formed in the burrow resulting from decomposing litter (15). For example, carbon sources including glucose, arabinose, and hydrocarbons can be metabolized by the dominating bacteria (*Bacillus*, *Rubrobacter*, and *Sphingomonas*) in active burrows (Fig. 6).

Desert sand is generally characterized by a deficiency of nitrogen and phosphorus (7, 16, 17). Previous studies have shown that kangaroo rat burrows contain more total nitrogen and organic matter content than in adjacent sand (3, 5, 6). Nitrogen-fixing bacteria like *Bacillus* and *Microvirga* were among the most prevalent genera in the active burrow community. Other nitrogen-fixing bacteria including *Azotobacter*, *Clostridium*, *Mesorhizobium*, and *Kribella* were found in both active and abandoned burrows, whereas none were detected in surface sand. Notably, *Azotobacter* and *Clostridium* were also present in anal swab, cheek pouch, and mouth samples. Nitrifying bacteria *Nitrospira* and *Nitrosospira* in active burrow community were found to be significantly more abundant than surface sand. Predictive functional pathway analysis further revealed significantly higher abundances of genes coding for nitrogen metabolism pathways within the active burrow community. The occurrence of nitrogen-fixing and nitrifying bacteria, along with genes coding for nitrogen metabolism pathways may be attributed to the accumulation of kangaroo rat excreta remains, leaf litter, and decomposition of seeds such as those from creosotebush (*Larrea tridentata*), within the burrows (18). The activities of kangaroo rats in the burrows increase soil nitrogen content and increase the population of bacteria contributing to the nitrogen cycle in burrow sand (19, 20). Additionally, the cellulolytic genera of *Cytophaga* and *Cellulosilyticum* found only in active burrows could be introduced into the burrows with the cached plants and seeds (Fig. 6).

Phosphorus solubilizing bacteria, such as *Azotobacter*, *Bradyrhizobium*, and *Flavobacterium*, were more abundant in the active burrow community than surface sand. Phosphate-solubilizing bacteria such as *Sporosarcina* and *Paenibacillus* were among the most abundant genera, with significantly higher levels observed compared to those in surface sand. Higher abundances of phosphate-solubilizing bacteria are consistent with burrows having higher nutrient availability due to plant debris, seeds, and feces (Fig. 6). Additionally, burrowing activity disturbs the sand, potentially mixing and redistributing nutrients, including phosphorus, which may enhance phosphorus availability.

Kangaroo rat burrows provide a stable internal microclimate where microbial taxa, less tolerant of high temperatures and high fluctuations, could survive. Temperatures within active burrows were measured at 25°C during the study using an infrared thermometer (Westward, model 28AF72). Although the Sonoran Desert temperature on the sand surface was 40°C conversely, which can often reach up to 50°C in the daytime and drop to 10°C at night (21). Bacteria, such as *Ferruginibacter* and *Mucilaginibacter*, only found in active burrows, have shown optimal growth at and below 30°C in previous studies (22–24). Moreover, within kangaroo rat burrows, favorable conditions promote the formation of biofilms and extracellular polymeric substances (EPS) by microbes, such as those produced by the *Mucilaginibacter* genus, known for its substantial EPS production. These biofilms create a favorable habitat for rare species, resulting in higher species diversity (25, 26). Previously, the higher diversity of microbial community in biocrusts compared to the underlying soil was attributed to biofilm formation (27).

The changes in bacterial composition resulting from the burrowing activities of kangaroo rats can influence plant composition and diversity. Previous studies have demonstrated that disturbances during burrow construction led to the mortality of certain plant species and the succession of others (28, 29). The increased availability of nutrient resources promotes the abundance of bacteria involved in nutrient transformations, such as ammonification, nitrification, and phosphate solubilization. These transformations lead to higher nutrient levels that can be utilized by plants. For instance, alterations in nitrogen content and water availability in kangaroo rat burrows facilitated the succession of plant species like *Eschscholzia mexicana* (5).

Although the measures of beta diversity did not statistically differ, most likely due to small sample sizes, NMDS and PCoA plots clearly differentiated the microbial communities belonging to active burrows and surface sand, which is consistent with our hypothesis. The presence of differentially abundant, unique, and distinct core microbial communities within active burrows could explain the microbial community shifts. The unique and more abundant genera (*Acidimicrobiales* and *Acidobacteria* Gp7), along with different core microbial communities (*Sporosarcina* and *Adhaeribacter)*, in active burrows indicate that these bacterial communities are well adapted to the specific environmental conditions in the burrow. Many strains of the core member *Adhaeribacter* are reported to be urease active, potentially contributing to the ammonification and regulating nitrogen and phosphorus content in the burrow sand (30, 31). Functional predictive analysis also demonstrated significantly higher abundances of genes coding for urea carboxylase and urease involved in urea metabolism in active burrow samples. Moreover, another active burrow core member (*Sporosarcina*) is related to phosphate-solubilization function (32) (Fig. 6). These genera may play important roles in shaping the microbial community structure and functioning within the burrow sand.

Subjection of kangaroo rat samples to 16S rRNA sequencing revealed that anal swabs and cheek pouch samples shared more genera with burrows compared to mouth and foot samples. All animal compartments contained many taxa unique to the animal, as well as many taxa shared with both burrows and surface sand—the former are likely animal-specific taxa while the latter are likely abundant generalist taxa. The overlap between burrows and kangaroo rat anal swabs and cheek pouches suggests that these animal compartments are likely inoculating burrows with these bacterial taxa through kangaroo rat feces and seed caches inside the burrows. Abandoned burrows exhibited greater variance in alpha diversity measurements, which might reflect differences in how long different burrows were abandoned.

It has been shown that the burrows of kangaroo rats remain stable for years after construction (2). Microbial communities in dryland soils can influence burrow stability by adsorbing on soil surfaces and producing EPS. The process improves soil strength, referred to as biocementation (33, 34). With the presence of nutrients and favorable physical conditions within the burrows, the active burrow microbiome may influence burrow stability through biocementation. Some of the unique genera found in active burrows, including *Ferruginibacter*, *Mucilaginibacter*, unclassified *Alcaligenaceae*, are known producers of EPS, while *Herpetosiphon* is known for filament production (26, 35, 36). Furthermore, *Bacillus*, *Pseudomonas*, and *Rhizobium* found in active burrows are reported to produce bioplastics such as *Polyhydroxyalkanoates* (PHA) (37–39). Other PHA producers like *Rhodococcus*, *Alcaligenes*, *Azotobacter* are identified in significantly higher abundance than surface sand (40, 41). It has been previously reported that accumulation of bioplastics may induce changes in soil stability by increasing microaggregate formations (42). These bacterial communities could be acting as gluing agents between sand particles through formations of filaments, EPS, and bioplastics, thereby contributing to the long-lasting stability of the burrows. The results of this study, therefore, highlight the need to further address the potential impacts of these bacterial communities on the mechanical properties of the burrows.

Our study focused on the bacterial composition of kangaroo rat burrows, kangaroo rats, and proximal surface sand in the Sonoran Desert in Yuma, AZ. It is likely that the microbial communities in these burrows differ from those in other locations and climates such as grasslands. We collected a limited number of samples to minimize disturbance to the natural state of the burrows. In addition, we faced challenges in trapping a large number of kangaroo rats within the limited time frame of the study, resulting in a limited number of kangaroo rat samples collected. Furthermore, we have limited information about how long the burrows we studied have been abandoned and the presence of other animals which may interfere with the microbiota. For example, snakes and spiders were observed inside the abandoned burrows during the study. Our study did not evaluate the potential influence of seasonal variations on burrow microbial composition. Extending the study duration and increasing the number of samples would provide a more accurate understanding of the variation in microbial composition through seasonal changes and the effect of disturbance. Despite these limitations, our study identified bacterial communities within burrows that are associated with kangaroo rat microbiomes and shows possibilities for diverse taxa that may play key roles in nutrient transformations and biocementation. The study represents a key step in understanding the relationships between animals and bacterial communities and how these interactions can shape their shared environment.

## MATERIALS AND METHODS

### Study site

The study site was chosen within the *D. deserti* habitat, which is situated in the sand dunes of the Sonoran Desert located in the Barry M. Goldwater Range in Yuma, AZ in the west public areas of Blocks A, B, and C (Marine Corps). This specific region is referred to as the Yuma Desert because it is characterized by low sandy plains, extensive dunes, and scattered hills composed of highly eroded volcanic rock (43). The area is recognized for its minimal precipitation and notable daily as well as seasonal temperature variations. From 2018 to 2022, the region exhibited high aridity and high temperatures, with minimum and maximum temperatures recorded at 12°C and 49°C, respectively. The average precipitation during this period was 0.18 mm/year (44).

Kangaroo rat burrows were scattered around the field site. Burrows were classified by their state of occupation by kangaroo rats as active or abandoned. Active burrows were classified using the presence of recent kangaroo rat spoor, visible kangaroo rat footprints, and tail drags that were left behind. Furthermore, active burrows had a round tunnel ceiling structure and well-kept floor characteristics. Abandoned burrows were classified by the presence of spider webs, irregular tunnel shape, and straws in and around the burrow. No visible kangaroo rat burrows were identified 8.4 m away from the active burrow site, which was named as no-burrow site. This site was used to collect surface sand samples.

### Sample collection

**Fig 7:**
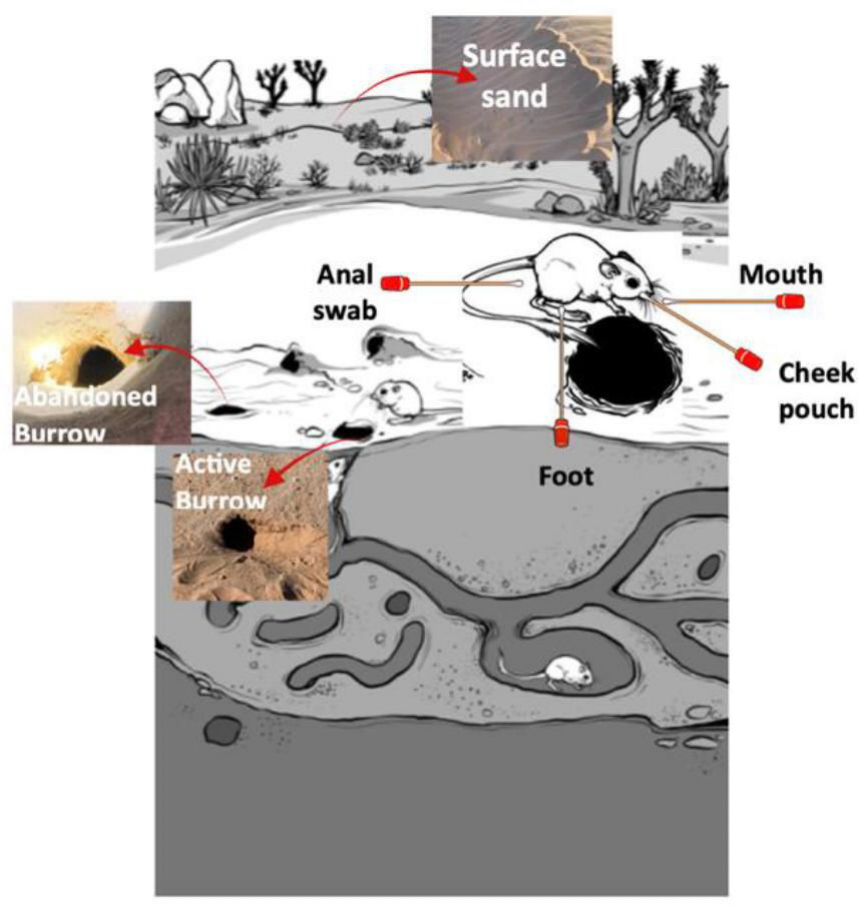
Depiction of microbial sampling locations from study site and from kangaroo rats (45). Active burrow: round shape of tunnel, well-kept floor characteristics indicate the active state of accommodation by kangaroo rats. Abandoned burrow: irregular shape, straws inside and around the tunnel indicate the inactive state of accommodation by kangaroo rats. The samples are taken from the burrow ceilings by scraping off the sand. Surface sand: collected from the no-burrow site where sand is loose, and no cementation can be observed. Rat mouth, cheek pouch, foot, anal swab.

Samples were collected during a 3-day period in late June 2022. Kangaroo rats were handled humanely under CSUS IRB Cayuse-21-22-3. No damage was made to the natural state of the burrows during the study. The ceilings of active and abandoned burrows were sampled by carefully scraping off the sand with a sterile spatula (SP Scienceware, cat. no. 4YMR9) and collecting in 50 mL sterile polyethylene conical centrifuge tubes (Thermo Scientific, cat. no. 339653). Loose sand samples were collected from three different active burrows, three distinct abandoned burrows, and surface sand from three distinct locations (Fig. 7). Individual *D. deserti* kangaroo rats were sampled by trapping them an hour before sunset. A total of four kangaroo rats were trapped in the study. Sample replicates from their mouth, cheek pouch, foot, and anal swabs were collected within an hour of sunrise the following morning. A total of four mouth samples (*n* = 2 from kangaroo rat 1 and *n* = 2 from kangaroo rat 3–4) were collected by inserting sterile cotton swabs (FisherBrand, cat. no. 14-907-12) straight into the mouth. A total of seven cheek pouch samples were collected by swabbing the sides of the mouth (cheek pouch) in circular motions. These included *n* = 3 from kangaroo rat 1, *n* = 2 from kangaroo rat 3, *n* = 1 from kangaroo rat 2, and *n* = 1 from kangaroo rat 4. Foot (*n* = 3, from kangaroo rats 1–3) and anal swabs (*n* = 3, from kangaroo rats 1–3) were sampled by swabbing as well. After sampling, the individual kangaroo rats were released unharmed. In total, three kangaroo rats were sampled. The microbial samples were preserved in a water-tight container and cold-stored on ice until they were transported back to the laboratory. The sand-filled centrifuge tubes were then preserved at −80°C to slow down microbial and biological activity before DNA extraction and sequencing.

### DNA extraction and sequencing

Prior to the DNA extraction process, kangaroo rat swab samples were pre-treated. First, the tips of the swabs were cut and placed into microcentrifuge tubes. Then, 500 µL of Lysis Buffer ATL (QIAGEN, Hilden, Germany) and 20 µL Proteinase K (Darmstadt, Germany) were added and incubated at 56°C for 10 min (46). The resulting 250 µL of microbial suspension were then used to extract DNA. DNA was extracted using E.Z.N.A Soil DNA Kit (Omega Bio-Tek, cat. no. D5625-01) according to the manufacturer’s protocol (47). The sand samples were not subjected to pre-treatment. Rather, DNA extraction was performed directly using the MagAttract PowerSoil DNA KF Kit (QIAGEN cat.no. 27000-4-KF) with the Kingfisher Flex DNA extraction machine following the Earth Microbiome project’s protocol (48). Additionally, to evaluate cross-contamination during DNA extraction, a negative control was added to the extraction plates.

The V4 region of the 16S rRNA gene was amplified using 515F: 5′-GTGCCAGCMGCCGCGGTAA-3′ and 806R: 5′-GGACTACHVGGGTWTCTAAT-3′ primers (49). PCR reactions were performed with DreamTaq DNA Polymerase (Thermo Scientific, cat. no. EP0705) using thermal cycler (Bio-Rad, USA). The thermocycler conditions consisted of an initial denaturation, 95°C for 3 min, followed by 30 cycles with denaturation at 95°C for 45 s, annealing at 50°C for 60 s, and extension at 72°C for 90 s. The final extension step was programmed at 72°C for 10 min, and a hold step was programmed at 15°C to keep the DNA stable until it is taken out. The PCR products were subjected to agarose gel electrophoresis using a 2% agarose gel in TBE buffer. The expected amplicon size of 300 bp and absence of amplification in the PCR negative control reaction were verified. The extracted DNA was then shipped to the Research Technology Support Facility Genomics Core at Michigan State University for DNA library preparation and Illumina amplicon sequencing of the V4 region of the 16S rRNA gene using the MiSeq V2 Standard platform producing 250 bp paired-end reads.

### Data analysis

FASTQ files were generated by the service provider. The generated files were used for data analysis with mothur v.1.48.0 (www.mothur.org) software package (50). Sequence analysis was performed following the MiSeq SOP (http://mothur.org/wiki/MiSeq_SOP) and the established procedures as previously described (50). Briefly, forward and reverse reads were aligned. At the beginning, a total of 204,348 sequences, all of which were unique, were obtained. Sequences with ambiguous base calls and homopolymers over 8 bp long were removed, and those exceeding the maximum sequence length of 275 bases were removed. Identical sequences were simplified and pooled together. Next, sequences were trimmed to target the V4 region of the 16S rRNA gene. The filtered sequences were then aligned to the SILVA (v138.1) reference database in the same positions, and non-informative columns were removed (51). Overhangs in the alignment were removed using filter.seqs. Next, sequences were preclustered allowing for up to two differences. Any chimeras formed during PCR were removed using the UCHIME tool implemented on mothur v.1.48.0 to avoid any false impression of diversity (52). Taxonomic classification was performed using Ribosomal Database Project 16S rRNA trainset 18, and taxonomies were assigned using naïve Bayesian classifier with 80% bootstrap confidence threshold (10).

Additionally, sequences identified as *Archaea (Eukaryota),* chloroplast, and mitochondria were filtered out. After quality filtering, the data set was reduced to 18,048 unique sequences and a total of 166,557 sequences across all samples. Both negative and positive controls were included in the sequencing set. Finally, after creating the shared file, ASVs were assigned using the classify.otu command with the final list, count table, and taxonomy files. The plots were created using RStudio version 2023.09.1+494. The relative abundance was calculated at the sample level by dividing the count of each ASV within a sample by the total count for that sample. Taxonomic levels (e.g., genus and phylum) were then filtered. Within each sample and group (e.g., active burrow, abandoned burrow, and surface sand), the relative abundances of identical taxa were combined. The mean relative abundance (%) was then calculated for each taxon across samples within each group. Any phylum/genus that had the maximum abundance of less than 3% were pooled together and labeled as “other” and plotted in a stacked bar chart using the ‘tidyverse’ package. The alpha diversity Shannon diversity and Inverse Simpson indices were determined using mothur. For beta diversity, Bray-Curtis distance measurements and non-metric multidimensional scaling (NMDS) were performed on mothur using the dist.shared and nmds commands. NMDS plots were then generated on RStudio using the “tidyverse” package. Differential analysis of ASVs was conducted using the algorithm described in “Metagenomic biomarker discovery” with linear discriminant analysis effect size (LDA) effect size (LefSe) in RStudio with “tidyverse” and “broom” package (53). The identification of significantly higher ASVs among sampling groups was accomplished using the Kruskal-Wallis sum-rank test. *P* values were adjusted by Benjamini-Hochberg correction. ASVs that showed significant differences (*P* < 0.05) among active burrow and surface sand samples were analyzed (54). For the analysis of overlapping taxa across different groups of samples, taxa at the genus level were analyzed, and Venn diagrams were generated using an online Venn diagram tool (https://bioinformatics.psb.ugent.be/webtools/Venn/). The core bacterial microbiome was detected using the “get.coremicrobiome” function on the mothur platform. Core microbiome components were found by detecting the ASVs being present in 100% of the tested samples from the same sample group at or above the relative abundance of 0.1% (55). For functional predictions, functional tables based on ASV abundances from 16S rRNA data were used to visualize as heatmaps created in RStudio using the “tidyverse” and “pheatmap” packages (56).

## Data Availability

The data from this study have been deposited in the NCBI repository under BioProject accession number PRJNA1108336 and Sequence Read Archive (SRA) accession number SRP505975.
